# Investigating a Low-Cost Dryer Designed for Low-Cost PM Sensors Measuring Ambient Air Quality

**DOI:** 10.3390/s21030804

**Published:** 2021-01-26

**Authors:** Abdul Samad, Freddy Ernesto Melchor Mimiaga, Bernd Laquai, Ulrich Vogt

**Affiliations:** Department of Flue Gas Cleaning and Air Quality Control, Institute of Combustion and Power Plant Technology (IFK), University of Stuttgart, Pfaffenwaldring 23, 70569 Stuttgart, Germany; freddernesto@gmail.com (F.E.M.M.); bernd.laquai@ifk.uni-stuttgart.de (B.L.); ulrich.vogt@ifk.uni-stuttgart.de (U.V.)

**Keywords:** low-cost sensor, PM sensor, air quality sensor, low-cost dryer, humidity influence on PM, air pollutants, air quality monitoring

## Abstract

Air pollution in urban areas is a huge concern that demands an efficient air quality control to ensure health quality standards. The hotspots can be located by increasing spatial distribution of ambient air quality monitoring for which the low-cost sensors can be used. However, it is well-known that many factors influence their results. For low-cost Particulate Matter (PM) sensors, high relative humidity can have a significant impact on data quality. In order to eliminate or reduce the impact of high relative humidity on the results obtained from low-cost PM sensors, a low-cost dryer was developed and its effectiveness was investigated. For this purpose, a test chamber was designed, and low-cost PM sensors as well as professional reference devices were installed. A vaporizer regulated the humid conditions in the test chamber. The low-cost dryer heated the sample air with a manually adjustable intensity depending on the voltage. Different voltages were tested to find the optimum one with least energy consumption and maximum drying efficiency. The low-cost PM sensors with and without the low-cost dryer were compared. The experimental results verified that using the low-cost dryer reduced the influence of relative humidity on the low-cost PM sensor results.

## 1. Introduction

### 1.1. Air Quality and Low-Cost Sensors

During the last years, the importance of good ambient air quality has strongly increased around the world. According to the World Health Organization (WHO), nine out of ten people live in places where the air quality guidelines are not fulfilled and every year poor air quality is related to 4.2 million premature deaths [1]. This is due to its relation to many negative effects on human health not only regarding respiratory diseases like deterioration of lung function, worsening of asthma symptoms, allergic reactions and airway obstruction [2], but also stroke, heart diseases and cancer [1,3,4,5]. Additionally, the impacts are not only related to human beings, but also to the ecosystems and earth climate system [6,7].

Considering these air pollution impacts, the air quality monitoring is nowadays of great concern, because it provides the necessary information to develop and implement suitable methods to improve ambient air quality. For this purpose, municipalities and authorities install stationary air quality monitoring stations in specific locations based on a measurement strategy and criteria for setting up the monitoring stations, e.g., height of sample inlets, distance to crossings, distance to roads, number of people impacted by air pollution, etc. [8]. These monitoring stations are usually equipped with devices, which are checked for accuracy in accordance with standards. These devices provide precise and reliable data about the air quality situation of the area. However, these instruments are in general costly and require highly trained professionals for their maintenance and operation [9]. Thus, only a small number of monitoring stations can be installed in a large area, which limits the spatial resolution. A good spatial resolution is necessary especially in areas where the pollutant distribution is not homogeneous, because of the influence of different sources as in the case of an urban environment [10].

In order to solve that issue, different citizen science groups and research institutes started investigation on using the low-cost sensors for this purpose available mainly for indoor air quality measurements and compared the results with the reference instruments [11,12]. These investigations helped to develop the low-cost sensors in a way that they could also be used for ambient air quality. Several companies have started to produce low-cost sensors, which are able to measure the air quality with lower expenses for operation and maintenance, allowing the possibility to deploy in large numbers and create a detailed air pollution map. Nevertheless, this new technology has the disadvantage that it is highly affected by different meteorological parameters, resulting in lower data quality [13]. Therefore, if a suitable solution to this problem can be found, then the low-cost sensors have the potential to work as a good support for the current conventional air quality monitoring stations [14].

### 1.2. Particulate Matter and Its Classification

The airborne Particulate Matter (PM) suspended in the atmosphere is formed by either natural or anthropogenic sources. PM is one of the most important pollutants when investigating air pollution due to its great impact on the environment and human health [15].

Airborne PM is a complex mixture of solid and liquid particles that can be:

(a) Primary, emitted directly into the atmosphere from either natural process such as windblown dust, smoke from forest fires, volcanic eruptions or anthropogenic processes such as automobile exhaust, smoke from power plants, etc.
(b) Secondary, formed by chemical reactions of gaseous components.

The transportation and environmental impact of PM depends on factors such as originating sources, composition and size of the PM. The classification based on size distribution can predict the residence time in the air as well as the transportation distance. In terms of health impacts, it can estimate the deposition intensity in the respiratory system. Hence, the air quality policies and emission regulations propose the PM limit values according to size fractions.

The PM size fractions are mostly represented as PM10, PM2.5 and PM1. PM10 are inhalable particles that may reach the upper part of the airways and lungs, while PM2.5 and PM1 are inhalable as well, but they can easily penetrate the lungs and perhaps might reach deeper parts of the lungs such as alveoli. The official limit values of PM are typically available for PM10 (coarse particles) and PM2.5 (fine particles), because in these fractions the PM is small enough to be inhaled and respired [16]. The impacts that PM can imply on the environment are diverse. The vegetation can be altered by the deposition of PM to the vegetated surfaces, which is mainly influenced by the PM size distribution and to a small extent on the chemical composition of the PM. Some of the effects caused by the PM can be abrasion, radiative heating and reduction of photosynthetic activity. In addition, the alkaline and acidic components may damage the surface or be absorbed through the cuticle [7].

**Figure 1:**
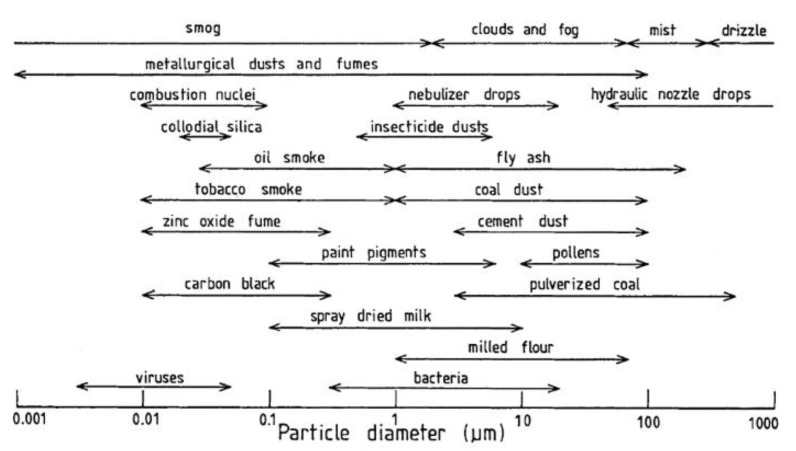
Particle size range for different airborne Particulate Matter (PM) [15].

In Figure 1, the most common PM and the diameter range in which they can be found is shown.

**Figure 2:**
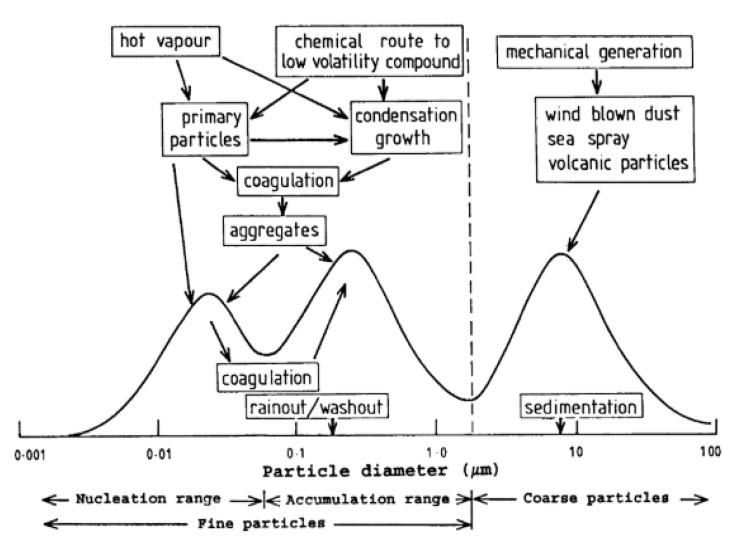
Schematic diagram of a typical size distribution and formation mechanisms for atmospheric particles [15].

It is very important to understand that the size, composition and concentration of PM strongly depend on the local activities, meteorological conditions and the PM itself. The size, number and chemical compositions can be transformed by several mechanisms until the PM is removed from the atmosphere. The PM is classified into three groups depending on the path they follow once they are formed as shown in Figure 2 [15].

(a) Nucleation mode (<0.2 µm diameter): Emitted from processes involving condensation of hot vapors or through gas to particle conversion in the atmosphere.
(b) Accumulation mode (0.2–2 µm diameter): These are grown from nucleation mode by coagulation or condensation of vapors.
(c) Coarse mode (>2 µm diameter): Formed by mechanical abrasion processes (soil dust, sea spray and many industrial dusts fall).

Fine particles are characterized by their etiology, their ability to remain suspended in the air and to carry material that is absorbed on their surface. The smaller the particle diameter, the longer it remains suspended in the air and the more hazardous it is [17].

### 1.3. Influence of Relative Humidity on Low-Cost PM Sensors

In many recent studies, it has been demonstrated that the low-cost PM sensors are highly affected when they work under humid conditions and overestimate the actual PM concentration [10,18,19,20]. The measurement principle for most of these low-cost PM sensors is light scattering principle with which the particles are counted. The number of particles is then converted to the PM mass concentration. The error occurs because the low-cost PM sensor counts not only the dry particles but also the wet water droplets that can happen at high levels of relative humidity and occur through condensation of the water vapor [19,20,21].

In 2018, Jayaratne et al. studied the behavior of the low-cost PM sensor model PMS1003 from the company Plantower. This low-cost PM sensor was tested against reference instruments in the laboratory and under field conditions. During the field experiments, there was fog formation in the early morning and the low-cost PM sensor considered these small water droplets as PM. Furthermore, the authors stated that a strong hygroscopic growth rate of PM mass was observed. The authors found that under high relative humidity levels, the low-cost PM sensors show a concentration 46% higher than the reference [19].

Akpootu and Gana presented the results obtained after the observation of hygroscopic growth on water soluble aerosols in 2013. The authors showed that at low relative humidity levels (below 50%) the aerosols do not show a significant increment in their size. However, at higher relative humidity levels, this effect is much more pronounced and can be expressed as an exponential curve [22].

Another recent study published regarding this topic was from the authors Brattich et al. that focused on two long-term measurement campaigns in order to compare the correlation between different low-cost PM sensors and reference instruments using different statistical approaches. In this publication, it was found that all low-cost PM sensors are highly affected during misty, cloudy and foggy conditions [10].

By keeping this problem in mind, the aim of this research was to develop and assess the performance of a low-cost dryer, which should be able to reduce or eliminate the negative effect of high relative humidity conditions on the ambient air quality measurements using low-cost PM sensors.

## 2. Measurement Technique and Methodology

### 2.1. Low-Cost PM Sensors and Reference Instruments

For the selection of the low-cost PM sensor for this research, previous studies performed by the authors and other researchers as well as the technical features and price of the low-cost PM sensors available in the market was taken into account. Numerous investigations were performed to compare different low-cost PM sensors with professional instruments in order to find the most suitable one for ambient air quality measurements [20,23,24]. Calibration models were also developed to improve the data quality obtained from these low-cost sensors [25]. The authors also tested well-known low-cost PM sensors such as SDS011 (Nova Fitness), PMS5003 (Plantower), OPC-N2 and OPC-N3 (Alphasense) to use them for ambient air quality measurements. OPC-N3 from the company Alphasense was chosen for this research as it showed better results compared to other low-cost PM sensors [26,27].

This low-cost PM sensor has a measurement size range from 0.35 μm to 40 μm sorting into 24 size bins. The time resolution of this low-cost PM sensor is one second and it provides a real-time histogram as well as the flowrate of the sampling air. This low-cost PM sensor also measures the temperature and relative humidity of the measurement chamber [28].

The reference instrument used to compare the results obtained from the low-cost PM sensor was an aerosol spectrometer, model EDM180 from the company Grimm Aerosol Technik Ainring GmbH & Co. KG. This device is able to measure the particle size range from 0.23 μm to 32 μm in 31 different size bins. It also measures ambient air temperature and relative humidity using a climate sensor. This device is equipped with a Nafion membrane-based dryer [29].

### 2.2. Experimental Setup

**Figure 3:**
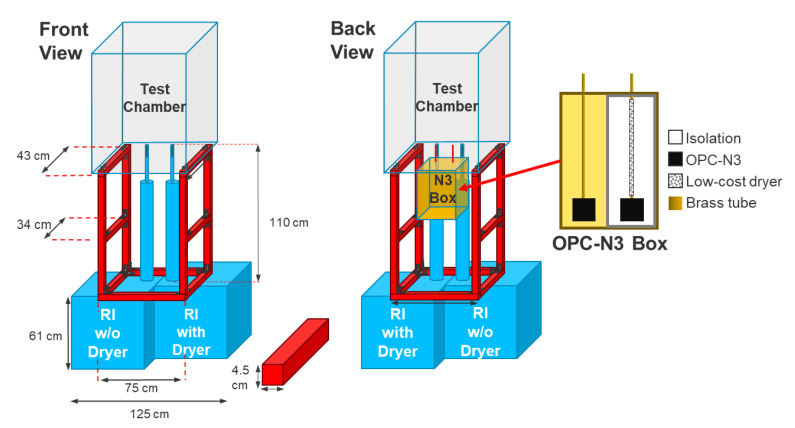
Experimental setup for laboratory measurements.

The experimental setup was designed to simulate the ambient air conditions on a laboratory scale. The experimental setup is shown in Figure 3. A test chamber was prepared, in which the measurements took place. The reference devices and the low-cost PM sensors along with particle generation and relative humidity control system were installed. The test chamber should be placed around 2 m above the ground as only the inlets of the reference devices and the low-cost PM sensors should be present inside the test chamber. For this reason, a metallic support that is shown in red color in Figure 3 was used to carry the test chamber. During the experiments, two low-cost PM sensors were installed in the test chamber. One of the low-cost PM sensors was equipped with a low-cost dryer (N3+dryer) and the other one without a low-cost dryer (N3). These low-cost sensors were compared with two reference instruments. One of these reference instruments was operated with a dryer (RI+dryer) and the other one without it (RI).

**Figure 4:**
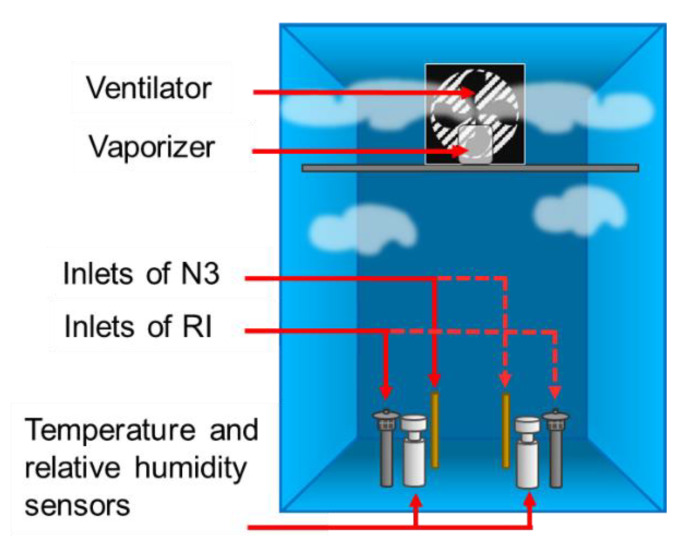
Test chamber configuration for experiments at different voltages.

The following equipment were placed inside the test chamber as it can be seen in the Figure 4.

- Vaporizer to simulate the humid conditions in the test chamber.
- Ventilator to homogenize the PM distribution inside the box.
- Inlet of the reference instruments and low-cost PM sensors.
- Temperature and relative humidity sensors.

**Figure 5:**
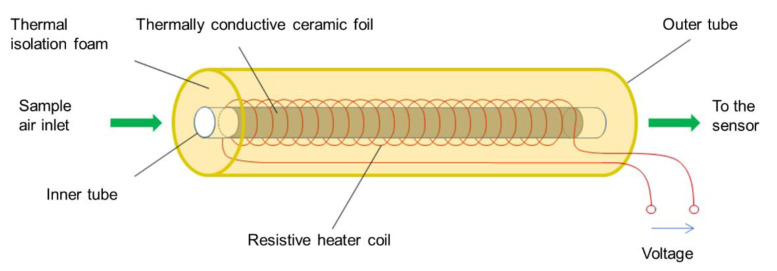
Schematic diagram of the low-cost dryer.

The low-cost dryer for the low-cost PM sensors was developed to reduce the relative humidity by heating the sampling air so that the influence of high relative humidity on the measured PM concentration can be reduced or eliminated. The low-cost dryer consisted of a thermally conductive brass inner tube on which winding of a metallic coil made from a resistive material such as constantan was done. A ceramic foil was put in between the inner tube and metallic coil to distribute the heat evenly throughout the inner tube. The heat is conducted through the inner tube to the sampling air. The thermally conductive properties of the ceramic foil allowed the proper heat transfer to the inner tube. The inner tube was insulated using an isolation foam to avoid any thermal loss and an outer tube was used to keep the low-cost dryer stable and to protect it from any mechanical destruction from outside. In Figure 5, the schematic diagram of the low-cost dryer developed during this research is shown.

The temperature of the low-cost dryer was controlled by adjusting the voltage applied to the metallic coil. For this system, a voltage range of 5 to 9 V was tested for this setup to find the optimum voltage for the operation of low-cost dryer. Using 5 windings per cm and 5-watt power on a 45 cm active surface of the dryer, it was possible to ensure a minimum of 5 volts required for the setup. The equations below show the corresponding formulas applied with a specific resistance of 0.97 ohms per meter for the metallic coil used in this project. The calculation results are shown in Table 1.

(1) $$R=V^{2}/P$$

(2) I=V/R

### 2.3. Methodology

This research was categorized in three steps. The first step consisted of setting up the test chamber for the experiments using the low-cost PM sensors with and without low-cost dryer and the reference instruments. Once the experimental setup was finalized, the second step was to find the optimum voltage to be used for the low-cost dryer. These experiments are further explained in Section 2.3.1. After finding the optimum voltage for the low-cost dryer, the third step was to check the performance and the efficiency of the low-cost dryer. Experiments were also performed to investigate the influence of low-cost dryer heating on the PM. In these experiments synthetic dust (Eskal14) from the company KSL Staubtechnik GmbH was used as particles and the experimental design was adjusted accordingly. These experiments are further explained in Section 2.3.2.

The experiments were carried out following a certain pattern containing different phases. This allowed to have a systematic analysis to test the performance of the low-cost dryer. The phases during the experiment were classified as following:

- Stabilizing phase: The phase in which the conditions inside the test chamber were allowed to stabilize after switching on the equipment.
- PM concentration increase phase: The phase in which the PM concentration was increased in the test chamber using a vaporizer and/or a particle distributor.
- Settling phase: The phase in which the PM concentration was allowed to settle after particle generation.
- Low-cost drying phase: The phase in which the low-cost dryer installed on one of the two low-cost PM sensors (N3+dryer) was activated.
- Final phase: The phase in which the low-cost dryer was switched off and the instruments were allowed to run for some more time.

#### 2.3.1. Experiments to Determine the Optimum Voltage for Low-Cost Dryer Operation

**Figure 6:**
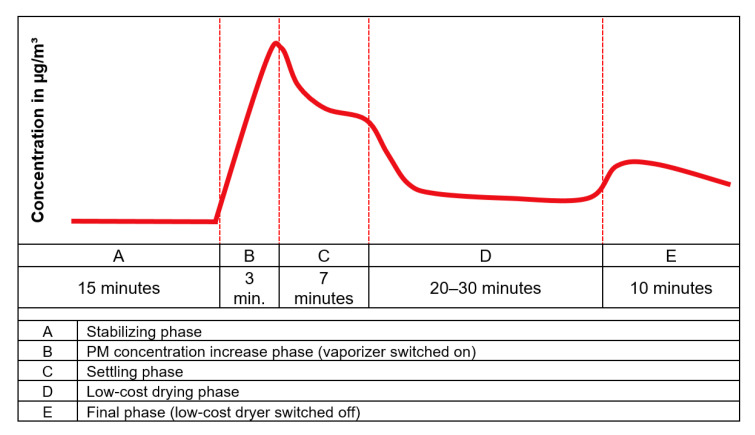
Temporal variation of an ideal PM concentration curve during a standard experiment.

The experiments to obtain the optimum voltage for dryer operation followed the pattern in which the stabilizing phase took place for 15 min. After this time, the vaporizer was switched on for three minutes, reaching a considerable PM concentration. The settling phase was seven minutes. Then the low-cost dryer installed at one of the low-cost PM sensors (N3+dryer) was switched on, while the other low-cost PM sensor was operated without the dryer (N3). The dryer was switched off after 20 to 30 min. In that way, it was possible to study and evaluate the effectivity of the low-cost dryer. The theoretical temporal variation of an ideal PM concentration curve during a standard experiment is shown graphically in Figure 6.

The above experiment was run at 6, 6.5, 7, 7.5, 8 and 9 volts. The optimum voltage was selected by comparing the ratio between the low-cost PM sensor without the low-cost dryer to the one with the dryer. This optimum voltage setting should be found to correctly dimension the low-cost dryer. A regulated thermal energy based on the sample air temperature and relative humidity could be used for the low-cost dryer operation.

#### 2.3.2. Experiments with Synthetic Dust

**Figure 7:**
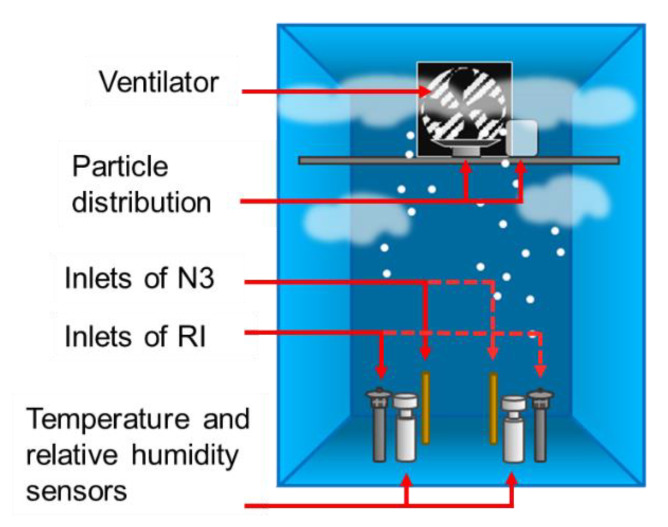
Test chamber configuration for the experiments with synthetic dust.

These experiments were conducted using synthetic dust with a particulate size distribution between 1 µm and 10 µm. The synthetic dust used for these experiments was a temperature resistant calcium carbonate “Eskal14”. This synthetic dust was chosen because of its narrow particle size distribution, excellent fluidity and suitability for wet applications [30]. The PM concentration generated during these experiments could not be calculated and hence it should be known only by means of the reference instruments. These experiments were performed using the optimum voltage applied to the low-cost dryer that was found in the previous set of experiments. The test chamber configuration for the experiments with synthetic dust is shown in Figure 7.

**Figure 8:**
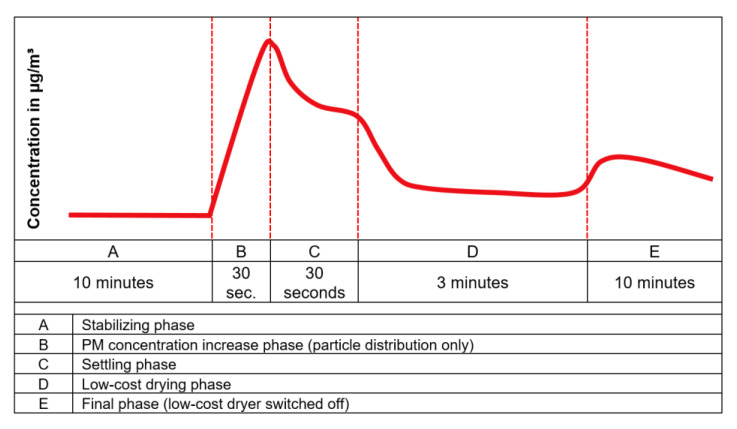
Temporal variation of an ideal PM concentration curve using synthetic dust only.

**Figure 9:**
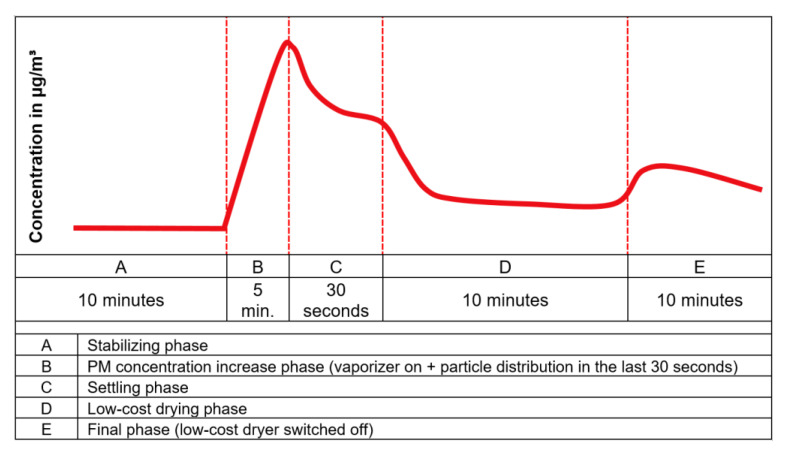
Temporal variation of an ideal PM concentration curve using synthetic dust and vaporizer.

The experiments with synthetic dust were divided in two parts. In the first part, the experiments were performed using synthetic dust only. In the second part, the experiments were done using both synthetic dust and the vaporizer. These set of experiments were compared with each other to observe the behavior of PM concentration with and without the addition of water vapors through the vaporizer in the presence of synthetic dust. The experimental pattern was similar to previous experiments. However, the particle distribution technique and the experimental time were modified. The duration of these experiments was different due to different settling time of the synthetic dust particles with and without the vaporizer. The temporal variation of the PM concentrations while using only the synthetic dust is shown graphically in Figure 8 and the temporal variation of the PM concentrations while using the synthetic dust and vaporizer is shown graphically in Figure 9.

## 3. Results and Discussion

In this section, the results obtained from the set of experiments mentioned in the previous section are shown.

### 3.1. Optimum Voltage for the Dryer Operation

**Figure 10:**
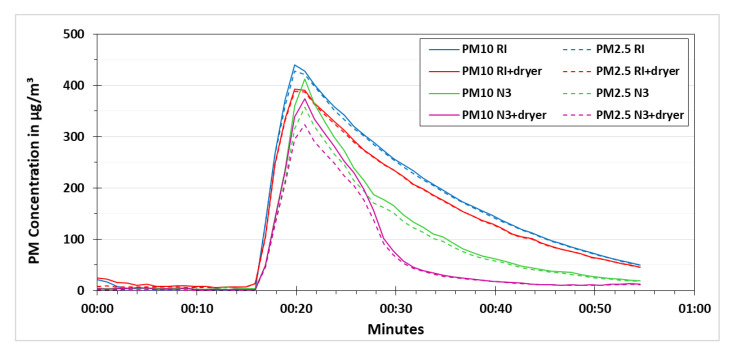
PM concentration comparison for testing the low-cost dryer at an applied voltage of 7 V.

**Figure 11:**
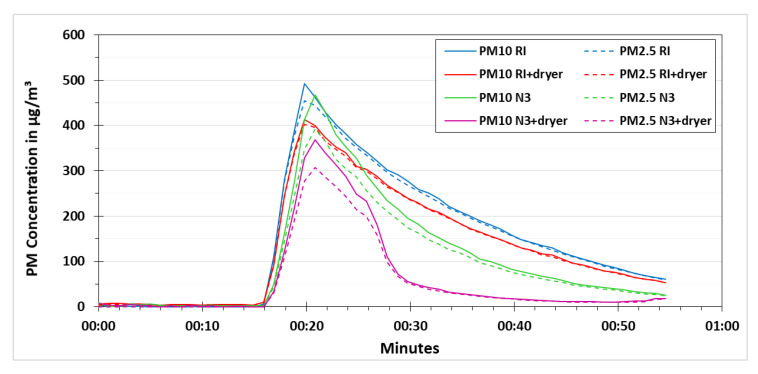
PM concentration comparison for testing the low-cost dryer at an applied voltage of 8 V.

**Figure 12:**
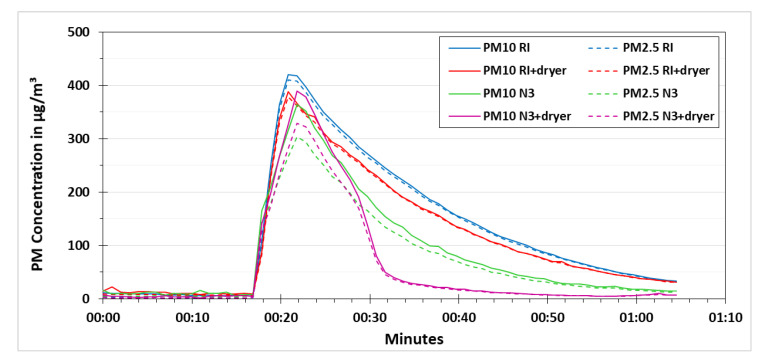
PM concentration comparison for testing the low-cost dryer at an applied voltage of 9 V.

As mentioned before, the heating of the low-cost dryer was controlled by applying different voltages. If the applied heating is less than required, then the low-cost dryer would not be able to efficiently remove the relative humidity effects on the results of the low-cost PM sensor. If the applied heating is more than required, then there is a chance that some part of the PM is evaporated that can lead to an underestimation of the PM concentration. By keeping that in mind, different voltages were tested during this research namely 6, 6.5, 7, 7.5, 8 and 9 volts. The results of 7, 8 and 9 volts are presented in Figure 10, Figure 11 and Figure 12, respectively. These experiments were performed according to the procedure explained in Figure 6.

In Figure 10, the experiment using the applied voltage of 7 V is shown. At the start of the experiment, the PM concentrations were allowed to stabilize in the test chamber for the first 15 min (stabilizing phase). The PM concentration measured by the low-cost PM sensors and the reference instruments at the end of this phase were below 10 µg/m³. After switching the vaporizer on, the PM concentration measured by the low-cost PM sensors as well as the reference instruments increased. It is noticeable that the PM10 and PM2.5 concentrations measured by the low-cost PM sensors and the reference instruments were similar. This indicates that the particles (water vapors) generated by the vaporizer were fine and the majority of these were below PM2.5 fraction. The peak PM10 and PM2.5 concentrations measured by the low-cost PM sensors and the reference instruments were in the range of 350 to 450 µg/m³ and 325 to 425 µg/m³, respectively. Even though a ventilator was used to distribute the particles homogeneously in the test chamber, still small variation in the PM concentrations measured by the low-cost PM sensors and the reference instruments was observed, which was assumed to be due to different inlet positions of these devices. The relative humidity was also increased in the test chamber during the operation of the vaporizer. After the vaporizer was switched off, the PM concentration measured by the low-cost PM sensors and the reference instruments started to decrease. The particles were allowed to settle before the low-cost dryer was switched on for one of the low-cost PM sensors. After around 25 min from the start of the experiment, the low-cost dryer was switched on for one of the low-cost PM sensors (N3+dryer). A significant decrease in PM concentrations can be observed for N3+dryer as compared to the low-cost PM sensor without the low-cost dryer (N3). At around 30 min from the start of the experiment, the PM concentration measured by N3 was almost double than the PM concentration measured by N3+dryer. The reference instruments were operated with and without the dryer from the start of the experiment as it was not possible to change the dryer settings for reference instruments during the experiment. The PM concentration measured by the reference instrument operating with the dryer (RI+dryer) was slightly lower than the PM concentration measured by the reference instrument operating without the dryer (RI). It is interesting to see that after increasing the PM concentration using the vaporizer, the PM concentration measured by RI+dryer had a similar concentration decline curve as the one measured by RI. It is assumed that since the reference instrument dryer works on a different principle (Nafion membrane) than heating to reduce the relative humidity, it does not instantly dry out the artificially generated particles using vaporizer. In the final phase, after switching off the low-cost dryer for N3+dryer at around 50 min from the start of the experiment, the PM concentration measured by N3+dryer increased slightly and nearly matched the PM concentration measured by N3.

The results obtained from the experiments performed for testing the other voltages had the same pattern. However, by increasing the applied voltage, the decline concentration curve for N3+dryer became steeper. Figure 11 and Figure 12 show the experiments using the voltage of 8 V and 9 V, respectively.

**Figure 13:**
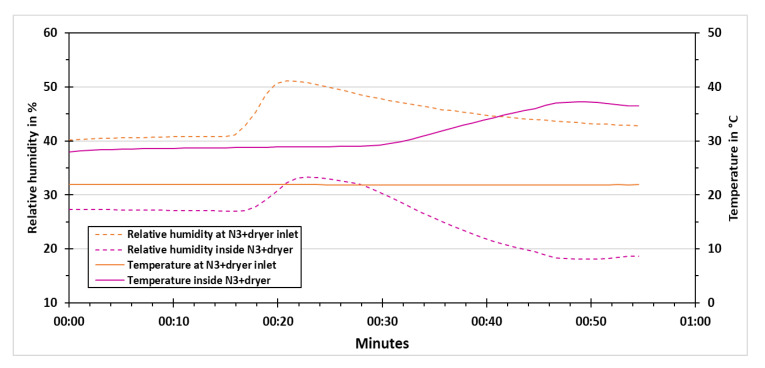
Temperature and relative humidity results for N3+dryer at an applied voltage of 8 V.

The behaviors of temperature and relative humidity were also observed throughout these experiments. These two parameters were measured at the inlet of the instruments using the climate sensor of the reference instruments. Apart from that, a temperature and relative humidity sensor is enclosed in the raw housing of the low-cost PM sensors. These measurements assist in understanding the performance of low-cost dryer during its operation. The experiments were performed at different temperature and relative humidity levels. As an example, the results of temperature and relative humidity for the low-cost sensor N3+dryer in the course of 8 V applied voltage experiment are shown in Figure 13. The temperature and relative humidity at the inlet of N3+dryer is shown in Figure 13 as solid orange and dashed orange lines, respectively. The temperature at the inlet of N3+dryer was constant at around 22 °C for the whole experiment. The relative humidity was marginally above 40% at the start of the experiment. A rise in relative humidity can be seen after 15 min from the start, when the vaporizer was switched on. For this experiment, the relative humidity peak was slightly above 50%. The vaporizer was switched off after three minutes of operation and the relative humidity started to decline after that. The temperature and relative humidity inside N3+dryer are shown in Figure 13 with solid pink and dashed pink lines, respectively. The temperature inside N3+dryer was somewhat below 30 °C that was moderately higher than the one at the inlet of N3+dryer while the relative humidity was lower compared to the N3+dryer inlet. This can be because of the working of electronics and mechanical parts inside N3+dryer. The increase in N3+dryer inside temperature can be observed after 30 min from the start of the experiment once the low-cost dryer is switched on. The peak temperature of approximately 37 °C was achieved at around 50 min from the start of the experiment. A slight decrease in this temperature is to be seen at the end of the experiment after switching off the low-cost dryer. The increase in N3+dryer inside relative humidity is also visible after the vaporizer is switched on. However, it is reduced considerably during the drying phase and it reached to a minimum value of below 20% which is even lower than the inside relative humidity of N3+dryer at the start of the experiment. After switching off the low-cost dryer, a minor increase in N3+dryer inside relative humidity was observed at the end of the experiment.

In order to understand the effectivity of the low-cost dryer, the ratios of PM concentrations measured by N3 and N3+dryer were calculated. Table 2 shows the results of the PM concentration ratios of N3 to N3+dryer at different voltages. These ratios were calculated when the low-cost dryer for N3+dryer was switched on during the low-cost drying phase. For a complete comparison, the ratios were obtained at three different points during the low-cost drying phase namely the start, mid and end of this phase. The results show that the average PM10 and PM2.5 concentration ratio of N3 to N3+dryer during the low-cost drying phase is in the range of 2 to 3 for the applied voltage of 6, 6.5, 7 and 7.5 V. There is a significant increase in PM concentration ratio for 8 V, where it reaches the value of around 4, which is the highest value for the tested voltages applied. Hence, the optimum voltage applied to the low-cost dryer for its operation was decided to be 8 V for further experiments using the synthetic dust.

### 3.2. Experiments with Synthetic Dust

These experiments were performed using the synthetic dust with the optimum voltage (8 V) for the low-cost dryer found in the previous experiments. These experiments were carried out using two different methods. The first method was to execute the experiment using only synthetic dust without using the vaporizer as mentioned before in Figure 8, while the other method included both synthetic dust and vaporizer that is explained in Figure 9. The settling time of the synthetic dust particles was much faster than particles produced by the vaporizer. Therefore, the duration of these experiments was reduced. These experiments lasted between 25 to 35 min.

**Figure 14:**
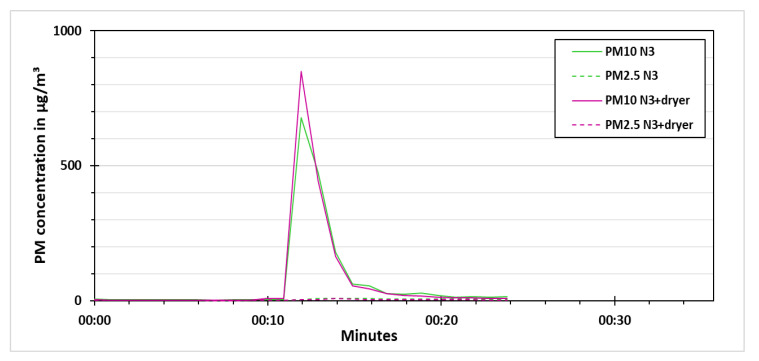
PM concentration for the synthetic dust experiment without vaporizer.

In the experiment without vaporizer, shown in Figure 14, the stabilizing phase was for around 10 min. After that, the particles (synthetic dust) were distributed in the test chamber for around 30 s. The synthetic dust particles were coarser than PM2.5 fraction. This is the reason that PM10 concentration measured by the low-cost PM sensors was increased during the particle distribution, while no change in PM2.5 concentration was observed during this phase. Since the PM10 concentration declined rapidly after the particle distribution, therefore the settling phase was very short for around 30 s. A momentary increase in PM concentration measured by the N3+dryer as compared to the N3 during the start of particle distribution was observed, which was assumed to be due to non-homogeneous particle distribution in the start. This momentary concentration difference disappeared rapidly in the settling phase. After that, the low-cost dryer was activated for N3+dryer for around 3 min. There was no significant difference of PM10 concentration measured by the low-cost sensors during the low-cost drying phase, which was expected as the relative humidity in the test chamber was not increased using vaporizer. This experiment showed that the particles were not destroyed from the heat of the low-cost dryer. It also showed that in the absence of water vapors, the N3 and the N3+dryer measured almost the same PM concentration. Hence, the low-cost dryer does not reduce the PM concentration in dry conditions.

**Figure 15:**
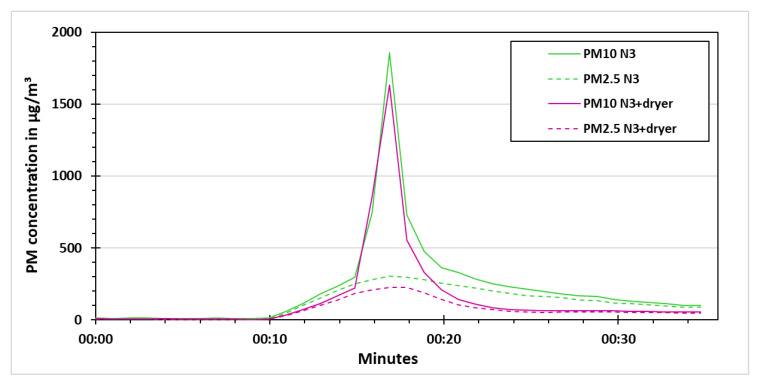
PM concentration for the synthetic dust experiment with vaporizer.

The same experiment was performed again with vaporizer for which the results are shown in Figure 15. The stabilizing phase was again for 10 min. In the PM concentration increase phase, the vaporizer was switched on for 5 min. The PM10 and PM2.5 concentration measured by the low-cost sensors increased to around 250 µg/m³ due to the vaporizer. In the last 30 s of this phase, the synthetic dust was distributed in the test chamber. This caused an increase in PM10 concentration for both the low-cost PM sensors in the range of 1600 to 1800 µg/m³. The particles were allowed to settle for 30 s. After that the low-cost dryer was switched on for N3+dryer for 10 min. The effect of low-cost dryer is evident from the PM concentration comparison for N3 and N3+dryer. The low-cost dryer swiftly dried out the particles (water vapors) from the vaporizer, while the synthetic dust particles remained that sedimented quickly due to gravity. After switching off the low-cost dryer, the PM concentration slightly increased as it was observed in the previous experiments with vaporizer.

## 4. Quality Assurance

Quality assurance was done to improve the reliability of the results obtained from the experiments. A comparison of the two reference instruments as well as the two low-cost PM sensors was performed before the experiments as a quality assurance. This assured that the results obtained from the reference instruments as well as the low-cost PM sensors are comparable to each other. The reference instruments and the low-cost sensors were tested by increasing the PM concentration using the vaporizer and then letting it settle. This procedure was repeated 10 times. A linear regression correction was applied to the data, which was then used for the whole experiments to have a valid comparison of the devices.

**Figure 16:**
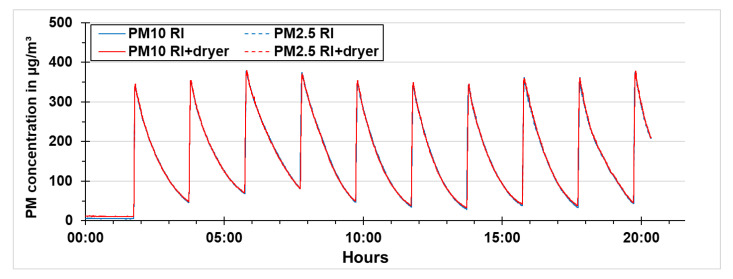
PM concentrations of the reference instruments during quality assurance.

In Figure 16, the results of PM concentrations of the reference instruments during quality assurance with the same dryer settings, i.e., dryer switched off, are shown. The particle size distribution during the quality assurance was below 2.5 µm. Therefore, the results of PM10 and PM2.5 are overlapping. It can be seen from the results that both the reference instruments follow the same pattern during the peak as well as during the fall of the PM concentration.

**Figure 17:**
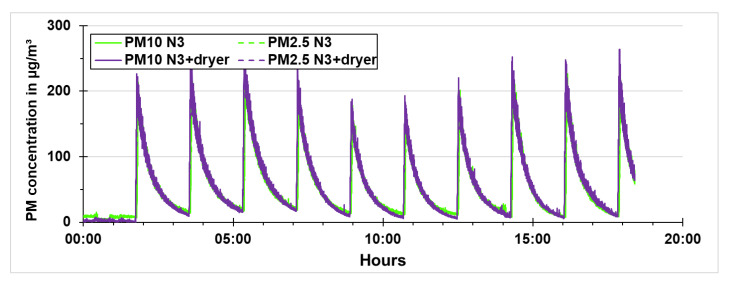
PM concentrations of the low-cost PM sensors during quality assurance.

The low-cost PM sensors were also tested in the same way as the reference instruments. In Figure 17, the PM concentrations of the low-cost PM sensors during quality assurance with the same dryer settings, i.e., dryer switched off, are presented. The results here are similar to the ones obtained with the reference instruments. The PM10 and PM2.5 concentrations are similar as the particle size distribution lies below 2.5 µm.

**Figure 18:**
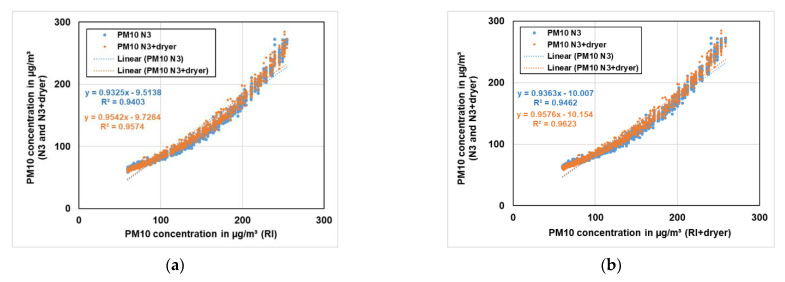
(**a**) Comparison of PM10 concentration of the reference instrument (RI) to the PM10 concentration of both low-cost PM sensors (N3 and N3+dryer) with the same dryer settings. (**b**) Comparison of PM10 concentration of RI+dryer to the PM10 concentration of both low-cost PM sensors (N3 and N3+dryer) with the same dryer settings.

**Figure 19:**
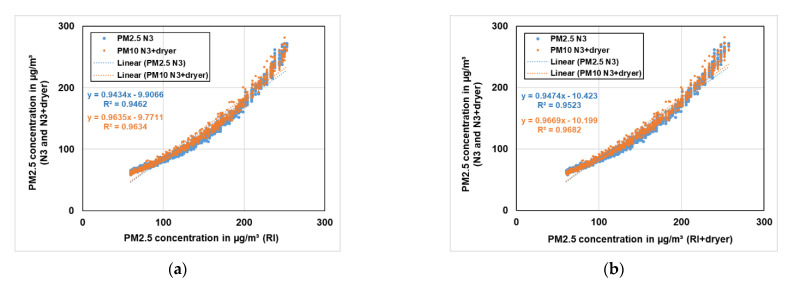
(**a**) Comparison of PM2.5 concentration of RI to the PM2.5 concentration of both low-cost PM sensors (N3 and N3+dryer) with the same dryer settings. (**b**) Comparison of PM2.5 concentration of RI+dryer to the PM2.5 concentration of both low-cost PM sensors (N3 and N3+dryer) with the same dryer settings.

In Figure 18 and Figure 19, the correlation of the reference instruments with the low-cost PM sensors for both PM10 and PM2.5 concentrations is shown. In Figure 18a,b, the comparison of PM10 concentration of RI and RI+dryer respectively to the PM10 concentration of both low-cost PM sensors (N3 and N3+dryer) with same dryer settings are shown. It can be seen that both low-cost PM sensors showed decent correlation of above 90% with the reference instruments (RI and RI+dryer) for PM10 concentration.

The PM2.5 concentrations were compared in a similar way. In Figure 19a, the comparison of PM2.5 concentration of RI to the PM2.5 concentration of both low-cost PM sensors (N3 and N3+dryer) is displayed, while in Figure 19b the comparison of PM2.5 concentration of RI+dryer to the PM2.5 concentration of both low-cost PM sensors (N3 and N3+dryer) with same dryer settings is shown. A correlation of around 95% was observed considering PM2.5 concentration for both low-cost PM sensors with the reference instruments (RI and RI+dryer).

## 5. Conclusions

It was concluded that the low-cost dryer is suitable for the application of measuring PM concentration using low-cost PM sensors. The low-cost dryer is able to eliminate the negative effects of relative humidity on the PM results measured by the low-cost PM sensors. The PM concentration comparison of the low-cost PM sensor with and without the low-cost dryer indicated that the low-cost dryer could dry out the water vapors generated from the vaporizer.

For operating the low-cost dryer with the low-cost PM sensor, the applied voltage controlled the heat applied to the low-cost dryer. The increase in applied voltage had a direct relation with the heat applied to the low-cost dryer. However, a significant increase of PM concentration ratio (PM concentrations measured by the low-cost PM sensor without the low-cost dryer to PM concentrations measured by the low-cost PM sensor with the low-cost dryer) was observed for the experiment with applied voltage of 8 V. This applied voltage helped to correctly dimension the low-cost dryer. A regulated thermal energy based on the sample air temperature and relative humidity could be used for the low-cost dryer operation.

The heat applied to the sample air through the low-cost dryer was adequate ensuring that there is no particle loss due to heating. No significant difference in the PM concentrations was observed by applying the heat and without it for the experiment with synthetic dust only, which indicated that low-cost dryer does not destroy the PM. The experiments with the synthetic dust demonstrated that the low-cost dryer should be suitable for measurements in real conditions.

The measurement technique and methodology presented in this research can be applied as it is or with some modifications to investigate the effect of the low-cost dryer on other low-cost PM sensors as a future work. The length of the low-cost dryer can be optimized for smaller platforms in order to make it more practical for field measurements.

## Figures and Tables

**Table 1:** Calculations for the low-cost dryer.

Tube			Winding			Wire	Resistance		Low-Cost Dryer		
Diameter (mm)	Length (cm)	Contour (cm)	Length (cm)	Density (cm)	Total length (cm)	Length (m)	Specific (Ω/m)	Total (Ω)	Power (W)	Current (mA)	Voltage (V)
8	50	2.5	45	5	225	5.65	0.97	5.5	5	952	5.2

**Table 2:** PM concentration ratios of N3 to N3+dryer at different voltages.

Low-Cost Drying Phase	6 V		6.5 V		7 V		7.5 V		8 V		9 V	
	PM10	PM2.5	PM10	PM2.5	PM10	PM2.5	PM10	PM2.5	PM10	PM2.5	PM10	PM2.5
Start	1.7	1.7	1.8	1.8	2.2	2.2	2.4	2.3	3.5	3.4	1.4	1.4
Mid	3.0	2.8	3.2	3.2	3.4	3.3	3.4	3.3	4.6	4.4	4.5	4.3
End	2.5	2.3	2.9	2.8	2.5	2.4	2.7	2.9	4.1	3.8	5.0	4.3
Average	2.4	2.3	2.6	2.6	2.7	2.6	2.8	2.8	4.1	3.9	3.6	3.3

## Data Availability

Not applicable.
